# Hedging Your Bets by Learning Reward Correlations in the Human Brain

**DOI:** 10.1016/j.neuron.2011.07.025

**Published:** 2011-09-22

**Authors:** Klaus Wunderlich, Mkael Symmonds, Peter Bossaerts, Raymond J. Dolan

**Affiliations:** 1 Wellcome Trust Center for Neuroimaging, University College London, London WC1N 3BG, UK; 2 Division of Humanities and Social Sciences, California Institute of Technology, Pasadena, CA 91125, USA; 3 Swiss Finance Institute, Ecole Polytechnique Fédérale Lausanne, 1015 Lausanne, Switzerland

## Abstract

Human subjects are proficient at tracking the mean and variance of rewards and updating these via prediction errors. Here, we addressed whether humans can also learn about higher-order relationships between distinct environmental outcomes, a defining ecological feature of contexts where multiple sources of rewards are available. By manipulating the degree to which distinct outcomes are correlated, we show that subjects implemented an explicit model-based strategy to learn the associated outcome correlations and were adept in using that information to dynamically adjust their choices in a task that required a minimization of outcome variance. Importantly, the experimentally generated outcome correlations were explicitly represented neuronally in right midinsula with a learning prediction error signal expressed in rostral anterior cingulate cortex. Thus, our data show that the human brain represents higher-order correlation structures between rewards, a core adaptive ability whose immediate benefit is optimized sampling.

## Highlights

► Humans learn interdependence of multiple environmental outcomes ► FMRI activity in insula pertains to trial-by-trial estimate of outcome correlation ► Correlation estimate updated by prediction error-based learning mechanism ► Subjects are able to use correlation information to make risk optimal choices

## Introduction

Risk is ubiquitous in nature with predation, starvation, adverse environmental change, or lack of reproductive opportunity acting as constant background variables that shape an animal's behavior. Animals evolved a variety of strategies to minimize risk such as diversifying mating behavior (Fox, 2003) or “bet-hedging.” For example, desert bees mitigate against large temporal variability in rainfall by stabilizing their birth rate (Danforth, 1999; Hopper, 1999). These risk-spreading strategies act to minimize between-year variance in reproductive success in a similar way to cost averaging, where financial investors periodically purchase risky assets to reduce the overall risk of an investment portfolio (Dodson, 1989). Our concern here is with risk as defined by outcome variability, measured from the variance of an outcome distribution. This is a first-order approximation of risk commonly used as a critical decision variable in ecological (Stephens, 1981) and financial (Markowitz, 1952) decision analysis.

Although the aforementioned strategies are naive with respect to higher-order structure in the environment, organisms can reduce risk even more effectively if they deploy knowledge of how different environmental states occur in relation to each other by representing correlations (Yoshimura and Clark, 1991). Thus, a lion learning that buffalo congregate at water holes on hotter days can reduce the chance of starvation by allocating more predation time to this food source by simply registering that the weather on a particular day is hot. In effect, knowledge of a covariance structure between discrete events allows inferences as to the presence, or in many instances quantity, of one outcome merely by observing a complementary event without actually having to sample on the inferred one.

Risk minimization is also a key concept in financial and insurance markets. Hedging, the process of combining multiple positions in different assets to reduce total risk in a portfolio is a common risk minimization strategy in financial investments (Jorion, 2009). Modern portfolio theory (MPT) (Markowitz, 1952) formalizes the concept of risk-spreading and relies upon correlations between multiple assets to specify how they can be most efficiently combined to maximize returns and minimize risk. Research in decision neuroscience provides extensive evidence for a neural representation of key decision variables (Doya, 2008) with a focus heretofore on value signals, putative inputs to the decision process such as action or goal values, and representations of expected outcome after a choice (Hampton et al., 2006; Knutson et al., 2005; Lau and Glimcher, 2007; Padoa-Schioppa and Assad, 2006; Plassmann et al., 2007; Samejima et al., 2005; Wunderlich et al., 2009, 2010). There is now good evidence that fundamental computational mechanisms underlying value-based learning and decision-making are well captured by reinforcement learning algorithms (Sutton and Barto, 1998) where option values are updated on a trial by trial basis via prediction errors (PE) (Knutson and Cooper, 2005; Montague and Berns, 2002; O'Doherty et al., 2004; Schultz et al., 1997). More recently, there is an emergent literature that suggests the brain not only tracks outcome value, but also uncertainty (Huettel et al., 2006; Platt and Huettel, 2008) and higher statistical moments of outcomes such as variance (Christopoulos et al., 2009; Mohr et al., 2010; Preuschoff et al., 2006, 2008; Tobler et al., 2009) and skewness (Symmonds et al., 2010).

An important component of outcomes, namely the statistical relationship between multiple outcomes, and what neural mechanisms might support acquisition of this higher-order structure has remained unexplored. In principle, there are several plausible mechanisms including the deployment of simple reinforcement learning to form individual associative links (Thorndike, 1911), or a more sophisticated approach that generates decisions based upon estimates of outcome correlation strengths. If the latter strategy is indeed the one implemented by the brain then this entails a separate encoding of correlations and corresponding prediction errors beyond that of action values and outcomes.

Here, we address the question of how humans learn the relationship between multiple rewards when making choices. We fitted a series of computational models to subjects' behavior and found that a model based on correlation learning best explained subjects' responses. Furthermore, we found evidence for a neural representation of correlation learning evident in the expression of functional magnetic resonance imaging (fMRI) signals in right medial insula that increased linearly with the correlation coefficient between two resources, a normalized measure of the strength of their statistical relationship. A correlation prediction error signal, needed to provide an update on those estimates, was represented in rostral anterior cingulate cortex and superior temporal sulcus. These behavioral and neural data provide evidence that subjects learn the correlative strength between rewards and are able to use this information to make risk-optimal choices.

## Results

**Figure 1:**
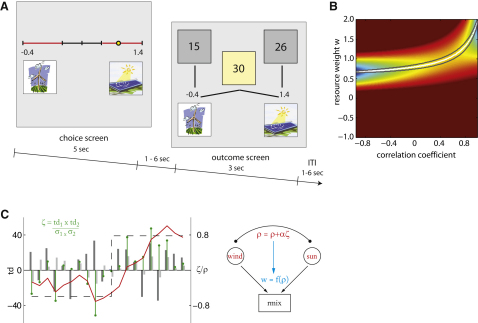
Experimental Design (A) Subjects were presented with a slider to set portfolio weights that determine the fraction of each resource (wind or solar power) in the energy mix (screen 1). The weights could be set within the range from −1 to 2, with a fixed relationship that both weights always add up to 1, i.e., w_wind_ = 1 − w_sun_. The trial outcome (screen 2) displayed the individual resource values for sun and wind, and the portfolio value of the combined mix (calculated by the weights from screen 1). (B) Optimal portfolio weight w_sun_ (w_wind_ = 1 – w_sun_) increases as a function of the correlation coefficient between sun and wind outcomes. The background color indicates portfolio standard deviation (blue = small SD, red = large SD). Optimal portfolio weights (for variance minimization) are displayed as white line, the gray lines indicate the 10% interval around the optimal choice (a deviation of that amount from the optimal weights would result in a 10% higher SD). (C) The correlation estimate ρ (red line) is updated from trial to trial (x axis) via a correlation prediction error ζ (green stems) and then in a second step used to allocate weights in every trial. Zeta is calculated as the cross-product between the two resource outcome prediction errors (gray bars). The correlation coefficient that was used to generate the data in this illustration is −0.60 during the first ten trials and afterward changes to +0.80 (dashed line). Learning of ρ from ζ is depicted here for a learning rate of 0.2.

To investigate how humans learn correlations between outcomes we scanned 16 subjects using fMRI while they performed a “resource management” game. This task invoked a scenario whereby a power company generates fluctuating amounts of electricity from two renewable energy sources, a solar plant and a wind park. We instructed subjects to create an energy portfolio under a specific goal constraint necessitating keeping the total energy output as constant as possible (Figure 1A). Subjects accomplished this by adjusting weights that determined how the two resources were linearly combined. A normative best performance is achievable by finding a solution that exploits knowledge of the covariance structure of these resources (Figure 1B), a task design that approximates a simple portfolio problem in finance. Importantly, the outcomes of the two resources covaried with each other and this correlation between the two outcomes changed probabilistically over time, requiring subjects to continuously update their estimate of the current correlation structure. This task is well suited for assessing subjects' estimate of the correlation strength because a good performance is only accomplished if subjects learn both the distribution of returns for each resource as well as their correlation. We rewarded participants according to how stable they kept the total output of their mixed energy portfolio relative to the variance resulting from an optimal strategy (specified by MPT-calculated optimal weights).

### Behavioral Model Comparison

We speculated that subjects might solve the task by learning the correlative strength between the resources via a correlation prediction error, calculated from the cross-product of the individual resources' outcome prediction errors (Figure 1C). This envisages that subjects represent a continuous measure of outcome correlation and update this metric on a trial-by-trial basis. To rule out alternative strategies we examined other computational models that could be used to guide choice in our task, and fitted the free parameters of each model to get model predicted portfolio weights that most closely resembled the actual responses for each subject.

One such alternative model-based strategy is to exploit trial-by-trial evidence to update a representation of the portfolio weights directly instead of first estimating the correlation coefficient. Similar to correlation learning, this model makes assumptions about the structure of the task and uses individual resource outcomes as a basis for learning. The main difference between the covariance based model and this model is that in the former, subjects update an estimate of the correlation via a prediction error and then translate this correlation strength into task-specific weights on every trial, whereas in the latter the estimates of task-dependent weights (i.e., the position on the response slider) are learned directly. This differentiation is important because the correlation coefficient is a normalized and therefore universal measure of the interdependence between the two outcomes, whereas appropriate mixing weights are task-specific and would need to be relearned if the variances of the individual outcome change or the goal of the task changes from risk minimization to maximization. Both of these strategies are model-based as they require an understanding of how the two individual outcomes interact. There are other potential modes of learning that we also consider. For example, subjects might implement a more simple model-free reinforcement learning based on Q-learning of action values for increasing or decreasing the weights. In contrast to the former approaches requiring subjects to attend to the individual resource outcomes, a subject who updates action values in this model-free way would instead consider the mixed portfolio outcome in every trial and try to minimize its temporal fluctuation using simple outcome based updating. Any change in behavior following a change in correlation between resources would then be due to a relearning of a new optimal mix of actions rather than a more complete knowledge of the structure of the environment. Finally, subjects might use a heuristic of detecting coincidences in the occurrence between outcomes, without a full representation of the strength of correlation.

**Figure 2:**
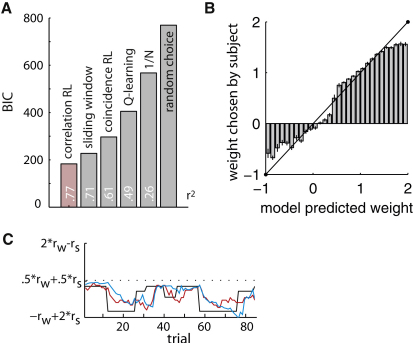
Model Fit and Behavior (A) The correlation learning model explained subjects' behavior best. Plotted are the Bayesian information criterions, which are corrected for the different levels of complexity in the models (smaller values are better). The r^2^ value represents the proportion of behavioral variance explained by each model. (B) Regression of actual weights on model predicted weights. Data is pooled over all subjects; for single subject results see Table 1. Note that the deviations at the extremes are a result from bounding the possible weight range at −1 and 2; any behavioral errors at the boundary could therefore happen only in one direction. Error bars = SEM. (C) Both the response of a representative subject (blue) and the model predicted weights (red) approach the normative best response under full knowledge of the generative correlation (black line) with some lag, which results from the time necessary to observe changes in correlation. Subjects responded after a 20-trial long observation-only phase (not shown).

Out of all tested models, the model based on tracking the correlation coefficient best predicted subjects' behavior (Figure 2A and Table 1). The weights estimated by this model match subjects' behavior very well, as shown by a comparison of model predictions and subjects' actual choices (Figure 2B) with the regression of actual observed weights on model predicted weights being highly significant in every individual subject (p < 0.0001; average R^2^ [standard coefficient of determination] across subjects = 0.77; see Table S1 available online). In fact, subjects' responses approximated normatively optimal portfolio weights while subjects attempted to keep the total energy output stable (minimize variance) (Figure 2C). Both model predicted and subjects' actual responses approach normatively optimal weights with some lag, the latter resulting from a need to have multiple observations to reliably detect any change in correlation strength. In effect, subjects' strategy of determining the correlation approximately compared to a normative calculation of the correlation coefficient over the outcomes of the past ten trials.

### Neural Representation of the Correlation Strength

**Figure 3:**
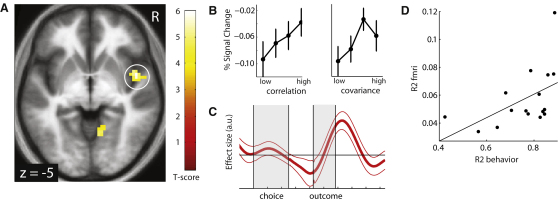
Neural Representation of Correlation Strength (A) Neural activity in midinsular cortex correlated with the trial-by-trial model predicted correlation strength between the two resource values at the presentation of the outcome screen. (B) Effect size plots (average percent signal change across subjects). Data plotted separately for trials in which the model predicted correlation strength was low and high in four bins (25/50/75/100 percentile of correlation range, errors bars = SEM). Activity in insula increased linearly with the correlation coefficient (that is, in contrast to the covariance, normalized by the standard deviations of the resources). Data were extracted using a cross-validation (leave-one-out) procedure to ensure independence of data used for localization and effect measure. (C) Time course plot of effect size for the correlation coefficient regressor. The correlation coefficient is represented at the time of the outcome screen, when new evidence becomes available, but not during the choice period. Thin lines = SEM. (D) Comparison of explained variance in the behavioral model with the explained variance in the fMRI analysis. Fluctuations in BOLD activity in midinsula can be particularly well explained within those subjects whose behavior is also well explained by the model (r = 0.50, p = 0.03). Each dot represents one subject and the line is the regression slope.

If the brain learns the relationship between two rewards by estimating their covariance then this predicts that we should observe a neural representation of the computations that support this process. Consequently, we tested for fMRI signals that track the covariance or correlation strength, and because the outputs vary, there should also be a signal that updates this information. Based on prior evidence, we predicted activity related to covariance would be seen in insular cortex or striatum, areas implicated in encoding the risk or variance of individual outcomes (Preuschoff et al., 2006, 2008). Consequently, we modeled subjects' trial-by-trial estimates of the correlation coefficient and regressed those model-predicted time series against simultaneously acquired fMRI data. We found BOLD activity in right midinsula varied with the correlation strength between the outputs of the solar and wind power plants (xyz = 48, 5, −5; Z = 4.12; p < 0.001 familywise error (FWE) corrected; Figure 3A). Right insula was the only region to survive cluster level whole brain correction and we provide a comprehensive list of all activated areas at a lower threshold (p < 0.001 uncorrected) in Table 2.

We next determined whether the correlation strength is represented either as covariance, a raw measure of how much the two variables fluctuate together, or as the correlation coefficient, a scale invariant metric of the covariance normalized by the standard deviation of each resource. We estimated two additional models using Bayesian estimation, with either the covariance or the correlation coefficient as parametric modulator, and compared the ensuing log-evidence maps in a random effects analysis. Activity in right midinsula was better described by the correlation coefficient than by covariance (exceedance probability of p > 0.999). The linear relationship between correlation coefficient and BOLD is visualized in a binned effect size plot (Figure 3B).

We then verified whether this signal was more strongly represented at the time of outcome, when new evidence is available to update estimates, or at choice when subjects actively readjust their allocated weights for the two resources (Figure 3C). In addition to plotting the effect time course we tested these neural hypotheses by estimating a design where the correlation coefficient acted as an unorthogonalized parametric modulator of activity at both the time of outcome and time of choice. In this analysis we observed significant effects of correlation strength solely at the outcome time (Z = 3.60, p = 0.01 FWE corrected) but not at the time of choice (Z = 2.40, p = 0.02 uncorrected).

If our behavioral model explains subject's choices and subjects' brain activity represents crucial decision variables in this process then we would expect that brain activity should be particularly well explained in those subjects in whom our model also provides a good choice prediction. This would be expressed in a relationship between the behavioral model fit and the model fit in the general linear model (GLM) against BOLD data. Consistent with our conjecture, we found a significant positive correlation between R^2^ in the behavioral model and R^2^ in the MRI analysis (r = 0.50, p < 0.03; Figure 3D). In effect, this confirms that our model explains a larger proportion of the fluctuation in the neuronal data in those subjects in which the model can also well explain choices.

### Neural Correlates of Correlation Prediction Errors

**Figure 4:**
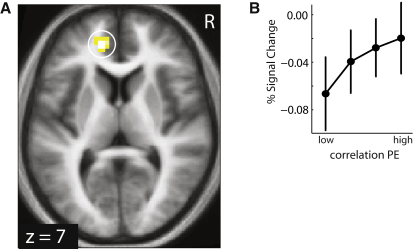
Neural Representation of Correlation Prediction Errors (A) Activity in rostral cingulate cortex correlated with the correlation prediction error. (B) Effect size plots (similar to Figure 3B) for the cluster confirm a linear effect.

A neural representation of correlation strength in our task entails that this estimate is updated over time, a process ascribed to a prediction error signal. Analogous to risk prediction errors for individual rewards (Preuschoff et al., 2008), the cross-products of the two outcome prediction errors provide a trial-by-trial estimate of the covariance strength. Using this regressor we found that a correlation prediction error was tracked in fMRI activity in left rostral cingulate cortex (xyz = −15, 44, 7; Z = 4.87; p < 0.003 FWE corrected; Figure 4 and Table 2).

### From Correlation to Portfolio Weights

**Figure 5:**
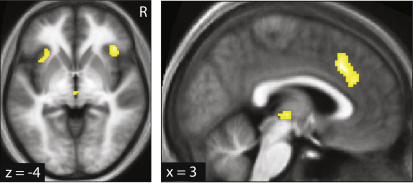
Absolute Weight Updates Activity in ACC/DMPFC and anterior insula correlated, at the time of the outcome screen, with the absolute amount that subjects update the resource allocation weights during the following choice.

After observing an outcome, participants may have an imperative to change the slider position if their currently set weights deviate from the estimated new best weights, in other words if they are suboptimal. We tested for a signal corresponding to the absolute (i.e., unsigned) deviation between current and new weights on the next trial and found corresponding BOLD activity in a region encompassing anterior cingulate (ACC)/dorsomedial prefrontal cortex (DMPFC) (xyz = 6, 26, 34; Z = 4.22; p < 0.001 FWE corrected) and in right anterior insula (xyz = 42, 23, −5; Z = 4.04; p < 0.04 FWE corrected) at the time of the outcome (Figure 5 and Table 2). In contrast, no areas corresponded directly to the portfolio weight values or a signed updating of weights, signals one would expect if subjects performed learning over task-specific weights instead of the correlation structure between outcomes.

Finally, an optimal solution to our task requires learning of the individual outcome variances in addition to learning the covariance structure. When we tested for neural activity coupled to local temporal fluctuations in the individual outcome variances we replicated previous findings in highlighting a neural representations of outcome risk in striatum (xyz = −18, 5, 10; Z = 3.81; p = 0.04 small volume corrected; Figure S3).

### Alternative Model Considerations

As an alternative to learning the correlation coefficient subjects might directly learn the weight representation and perform RL over the weights instead of the correlation coefficient. If that were the case then one would also expect to find a neuronal representation of the weights and weight prediction errors, which were conspicuously absent in our data. Another possibility could be that subjects simplified the problem to detecting outcome coincidences (both outcomes either above or below mean versus one outcome above and the other below mean) instead of fully quantifying the trial-by-trial covariance. In that case we would expect to find a neural signal pertaining to mere outcome coincidences. We found no activations coupled to either the weight or the weight prediction errors, or the trial-by-trial coincidences anywhere in the brain at our omnibus cluster level threshold of p < 0.05. Together with the inferior behavioral fit of the coincidence model this suggests that subjects quantified the trial-by-trial relationship between outcomes. We also implemented a model-free Q-learning algorithm as further alternative strategy, which was clearly outperformed by the correlation model.

## Discussion

We show that human subjects are adept at learning correlations between two dynamic variables, a process also represented neurally. Subjects were highly effective at exploiting this key metric of the statistical relationship between the two individual resources to guide choice in a task requiring minimization of outcome fluctuations. This finding is in contrast to an often-proposed model in behavioral finance, which suggests disregarding environmental structure and using fixed weights according to the 1/N rule (Benartzi and Thaler, 2001). Our subjects performed better than this simple heuristic and learned a more optimal strategy through repeated observations. At a neural level, fMRI signals in right midinsula were coupled to the current correlation coefficient, whereas activity in rostral anterior cingulate encoded a correlation prediction error, a signal used to update an estimate of the correlation strength based on new evidence in every trial.

Although learning individual outcomes is a central part of decision making, the availabilities of different rewards are rarely independent of each other in a natural environment. Our results provide evidence that subjects also learn the relationship between multiple outcomes by tracking their correlation, and this information can be used to decrease overall sampling risk. Commonly observed risk aversion in animals (Kacelnik and Bateson, 1996) and humans (Tversky and Kahneman, 1981) is rational in an evolutionary context, as a small but constant supply of food that always exceeds the critical minimum for survival is far more beneficial to viability than periods of alternating deficiency and extreme excess. In some other instances, risk-seeking behavior may occur, such as in gamblers, and may promote exploration and learning. Note, however, that also in that case a representation of the correlation in the environmental structure is beneficial, as this information can be used both for risk minimization or maximization.

To generalize our results to more natural situations, we have to ascertain that the findings reflect a specific mechanism of correlation learning instead of incidental task variables. Plausible possibilities include shortcuts such as learning the position on the response slider by a model-free gradient descent mechanism or using a model-based strategy, but without representing individual outcome variances and normalized correlation coefficients and instead directly learning a representation of the portfolio weights. Our behavioral and neural data render all these explanations very unlikely. The best-fitting learning rate for outcome variance is similar to the learning rate for correlation and significantly above the one for value for each individual subject. Importantly, we ensured that the signals in our study were not spurious reflections of the individual variances of solar and wind plant outputs by explicitly modeling these signals with additional (unorthogonalized) parametric regressors. A fluctuating trial-by-trial estimate of the outcome variance is also represented neurally in striatum (Figure S3), an area previously implicated in variance learning (Preuschoff et al., 2006). Although these neural signatures of risk and risk prediction errors were somewhat weaker compared to covariance signals, we suggest this observation is due to an amalgamation of signals tracking the two separate resource variances within the same area, and because the variance of the two outcomes fluctuated only slightly over the course of each experimental block. Importantly, we found no significant correlations with signals pertaining to alternative decision models anywhere in the brain at p < 0.05 corrected. Specifically, we examined if there was evidence for a direct representation of desired resource weights, or weight prediction errors, signals one would expect instead of the correlation coefficient if subjects used a more task-specific strategy. We also did not find significant correlations with a more qualitative measure of coincidences instead of fully quantified correlations. Together with a superior behavioral fit of the correlation learning model, this strongly supports the specificity of our neural results and effectively discounts the possibility that the observed activations here relate to incidental task related learning processes instead of learning the correlation between outcomes.

We found that anterior insula tracked the correlation strength between the outputs in a site slightly posterior to regions previously implicated in tracking variance (Mohr et al., 2010; Preuschoff et al., 2008). Combined, these findings suggest that insular cortex may support a general role in processing statistical information about the environment. At the same time, anterior insula has been implicated in representing bodily states and their translation into feelings and possibly awareness (Craig, 2009). Note that the calculus-like role proposed here does not contradict the idea that anterior insula represents subjective aspects of experience. Indeed, the somatic marker hypothesis postulates that rational decision theory requires emotional anticipation of outcomes (Bechara et al., 1997), such that seemingly prudent behavior and emotional decision making are intertwined (Paulus et al., 2003). The finding of a slightly posterior encoding of correlation relative to risk also tallies with a structural model for how unconscious state representations might be integrated into a sentient self along a posterior to anterior insula (Craig, 2009). Adequate emotional risk assessment is immediately relevant for fight or flight responses and might therefore require a more direct link to awareness then the meta parameters of how multiple such variables relate to each other (Bossaerts, 2010). The latter assessment is largely subconscious and may, as implicit function, also be enacted during low-level processing of multidimensional stimuli such as music and rhythm. Interestingly, such tasks have previously been associated with insula activation (Koelsch et al., 2006; Platel et al., 1997). Our data show that the brain encodes the correlation coefficient of two outcomes, a normalized value, instead of the covariance itself. In light of previous data (Bunzeck et al., 2010; Padoa-Schioppa, 2009; Seymour and McClure, 2008), this hints that scale invariance is a ubiquitous concept in encoding decision variables in the brain.

The representation of a prediction error in anterior cingulate fits neatly with mounting evidence that this area is involved in learning and behavioral control. Several previous studies report a role for anterior cingulate in an error-driven reinforcement learning system (Kennerley et al., 2006), and in prediction errors for actions (Matsumoto et al., 2007) or social value (Behrens et al., 2008). Together with risk prediction errors in anterior insula (Preuschoff et al., 2008), this teaching signal for correlation strength might belong to a broader system involved in learning the statistical properties of the environment.

We also observed an anticipatory signal reflecting an impetus to shift resource allocations on the next trial in order to keep the total energy output stable. Interestingly, this signal was expressed in a DMPFC cluster previously linked to updating learning in relation to environmental volatility (Behrens et al., 2007), implying a more general role for this region in adapting behavior to fluctuations in the statistical characteristics of the environment. Most task-modulated activity, including correlation strength, its prediction error, and a signal reflecting the need to alter responses, occurred at the time of outcome rather than at choice. This suggests that task-relevant computations, including an evaluation of the appropriate action to take after each outcome, occur at the point when individuals can best harvest new evidence. As we focused on the mechanism of learning the correlation strength, rather than on how subjects use this information, this raises the question of how exactly information about a covariance structure is applied in a natural sampling environment. Here, we instantiated this mapping of correlation coefficients into energy resource weights by using the normative function derived from MPT. We assume subjects learned the form of this nonlinear transformation during initial training, but it remains a question for future research how this translation is applied. Based on our present results and previous findings that the brain encodes other statistical parameters such as variance and skewness of outcomes (Preuschoff et al., 2008; Symmonds et al., 2010), we speculate that in more naturalistic environments subjects form structural representations of the world by encoding summary statistical parameters. Such a parameterized representation is both efficient and flexible: the optimal response is dependent upon three parameters—the magnitude, variance and correlation of the available resources—and knowledge of the individual parameters allows fast adaptation in light of changes to any one of them. One way to expand our research to more natural situations could be by changing the cost function to mimic an ecological survival game with perishable outcomes. Such a paradigm would allow one to determine if subjects indeed follow a variance minimizing strategy and incorporate information about reward correlations.

The recent financial crisis has amply demonstrated that even experts have difficulties regulating correlated risks in the financial domain and investors often deviate from rationality when making financial decisions (Daniel et al., 2002; Kuhnen and Knutson, 2005). In contrast, we show here that individuals are adept at detecting and responding to correlations and appropriately selecting actions to minimize risk in an intricate learning task. Indeed, this exquisite sensitivity taps into an adaptive and evolutionary conserved ability of implicit neurobiological systems to learn environmental reward structure through trial-by-trial sampling; intrinsic behavior that might even supersede that of financial experts deciding about explicitly described statistics.

## Experimental Procedures

### Subjects

Sixteen healthy subjects (7 female; 18–35 years old) with no history of neurological or psychiatric illness participated in the study. Two additional pilot subjects from the lab were excluded from the final analysis, as they were already familiar with the hypotheses in the experiment. The study was approved by the Institute of Neurology (University College London) Research Ethics Committee.

### Task

To investigate whether and how subjects learn the reward structure in the environment we designed a portfolio-mixing task in which knowledge of the correlation between two resource outcomes could improve performance. Subjects' task was to keep the combined output of two power stations as stable as possible (i.e., minimize the variance of an energy portfolio) by mixing the fluctuating outcomes of these two individual resources. They accomplished this by adjusting weights that determined how the resources were linearly combined. A normative best performance is achievable in this task by finding a solution that directly depends on knowledge of the covariance structure of these resources, a task design that approximates a simple portfolio problem in finance (Markowitz, 1952).

We presented the task to subjects as a resource management game that invoked a scenario whereby a power company generates fluctuating amounts of electricity from two renewable energy sources, a solar plant and a wind park. The resource outputs r_sun_ and r_wind_ were drawn as random numbers in every trial from distributions with a common mean M and variances σ^2^_sun_ and σ^2^_wind_. Importantly, the two outcomes covaried with each other, and the strength of this correlation changed probabilistically over time. This feature encouraged subjects to form an estimate about the mean and variance of the individual outcomes and continually update their assumption about the correlation strength.

Subjects participated in three consecutive experimental blocks, each corresponding to a 21 min long session in an fMRI scanner (Siemens Trio 3T). They were instructed that the correlation would probabilistically change over the course of the study but were not given further details about specific parameters used. We also told subjects that the mean and variance of the two resources would remain constant over one block of the experiment, a simplification to an otherwise quite complex task that enabled subjects to perform well within the settings of this experiment. As our goal was to assess covariance learning (in contrast to learning the values and risk) this did not adversely impact on any mechanism we wanted to observe. However, mean and variance values were different for each block. To give subjects the opportunity to learn these basic statistical parameters (mean and variance) before making portfolio choices, we presented them with a 20-trial observation phase at the beginning of each session. In this phase, which immediately preceded the start of fMRI data acquisition, subjects only observed the individual outcomes of the two resources and did not make any choices. There was no change in the ground truth correlation during this phase. Data from pilot studies and model simulations confirmed that 20 observations of a time series were sufficient to form an estimate of its mean and variance. The observation phase was followed by 84 choice trials, consisting of a 5 s choice period and a 3 s outcome period, separated by a blank gray screen of 1–6 s duration (uniform distribution). The intertrial-interval was also 1–6 s (Figure 1A).

The portfolio weights (w_sun_, w_wind_) indicate how much of a fraction the portfolio contains from both resources r_sun_ and r_wind_ (portfolio outcome value V_p_ = w_sun_^∗^r_sun_ + w_wind_^∗^r_wind_). Subjects were allowed to set the portfolio weight w_sun_ within a range between −1 and 2. Setting negative weights allowed subjects to trade-in a fraction of the trials output from one resource in exchange for multiplying the other output by the same fraction. This concept echoes the possibility of short selling in financial markets and is important for this task as it permits risk minimizing for positively correlated resources (see the section on variance minimizing strategies in the Supplemental Information for further details). The constraint that both weights always add up to 1 automatically determined the weight of the other resource (w_wind_ = 1 − w_sun_). A horizontal line on the choice screen represented the slider during the choice period and icons of a solar and wind plant on both ends indicated which resources were mixed in the portfolio. The parts of the slider involving a negative weight were red and the middle part with both positive weights was shown in white with the center position corresponding to a mix with equal weights. A yellow dot on the slider indicated the current position and portfolio weights were additionally shown numerically next to the resource icon. Subjects were able to make responses during the entire 5 s choice period by pressing two buttons on a button box with their right hand. Each button press moved the current slider position a discrete step of 0.1 units in either direction. Moving the slider a step toward the right always increased the weight for sun and decreased the weight for wind. A new choice period started with the portfolio weights from the last trial and subjects were allowed to freely move the slider as many steps in either direction as they wished during the choice period. Importantly, subjects always had to determine the weights for the current trial prior to seeing the actual outcome. Due to inherent stochastic outcomes, and because serial outcomes were independently drawn, the only rational strategy was to set the weights in a way that would yield the least portfolio variance in the long run and this measure depended on the current correlation.

To determine subjects' performance we benchmarked their portfolio fluctuation against the fluctuation of a portfolio with optimal weights. The normative solution was calculated by the risk minimizing formula of portfolio theory (see Supplemental Information for details). This ensured that subjects were fairly scored given the stochastic outcomes on a trial-by-trial basis (i.e., even if subjects played optimally the portfolio outcome would fluctuate around the target with the amount of fluctuation dependent on the current covariance). Subjects received reimbursement of 10£ flat plus a fraction of the maximum bonus of 45£ in relation to task performance (Table S1). All participants received basic instructive information about hedging strategies (similar to the Supplemental Information variance minimization strategies and Figure S2) and practiced the task (same number of trials than in the fMRI study but with different parameters for outcome mean and variance) on a separate day prior to scanning. Note, however, that all instructions concerned exclusively how to set portfolio weights (i.e., how to respond) but not how to learn correlations itself. Therefore this latter process cannot be confounded by the explicit information given here. The reason for using a seemingly intricate portfolio task over having subjects merely report the correlation directly is that explicit assessments of decision variables by self-report are often biased (Kagel and Roth, 1997). Our procedure is in this respect very similar to other commonly used behavioral measures such as auction bidding (Becker et al., 1964; Plassmann et al., 2007) to identify subjects' unbiased value preference. Another advantage of our task is that response behavior does not depend on individually subjective valuation or risk preference. Performance and payout were only related to how close subjects' behavior matched the normative optimal solution (thereby incentivizing an accurate correlation representation) but was independent of the actual amount or variance of the produced energy mix.

Importantly, during the experiment subjects never received direct feedback on their performance at minimizing energy fluctuations (i.e., only saw trial-by-trial outcomes) and the bonus and optimal weights were only revealed after the experiment. We omitted feedback during the task to prevent subjects from using a strategy that is based on optimizing the performance feedback instead of learning the correlation of the individual outcomes. Although the portfolio value is shown on every trial, and the deviance of this value from its mean gives some hints to performance, this is only a crude measure of whether the current weights are good because even with optimal weights the amount of portfolio fluctuation depends on the current correlation.

Because the optimal mixing weights (portfolio weights) in our task depend on individual variance from solar and wind power plants and their correlation strength, the best strategy is to learn the variances and correlations by observation of individual outcomes and then translate these estimates into an optimal resource allocation (i.e., weightings). Although subjects could learn the statistical properties underlying outcome generation by observation, the outcomes of individual trials were unpredictable. Their task was then to continuously mix the two resources into an energy portfolio and thereby minimize the fluctuation of the portfolio value from trial to trial.

### Generation of Outcome Values

Both resources fluctuated around a common mean, with outcomes drawn from a rectangular distribution with a specific variance. In our task the standard deviation of one resource was always twice that of the other because this maximized the influence of the correlation on the portfolio weights (see Figure S1 for details). The sequence of correlated random numbers for the two resources were generated by the Cholesky decomposition method (Gentle, 1998). This was realized by first drawing random numbers x_A_ and x_B_ for resources A, B from a rectangular distribution. The outcome of the second resource x_B_ was then modified as x_B_ = x_A_^∗^ r + x_B_^∗^ sqrt(1 − r^2^), whereby r is the generative correlation coefficient. Finally, x_A_ and x_B_ were normalized to their desired standard deviations (in the three blocks: 20/10, 15/30, 10/20) and common means (30, 50, 40). We chose a rectangular distribution to increase the sensitivity of our fMRI experiment in finding neural correlates of covariance and covariance prediction errors as the linear regression against BOLD activity is most sensitive if the values of the parametric modulators are distributed along their entire range. This is not true for normal distributed outcomes, which have proportionally the largest amounts of data close to the mean.

We varied the generative correlation strength in discrete steps of −0.99, −0.3, 0.4, 0.7, 0.95, and 0.999. The observable correlation through sampling by the subject will, however, very on a continuous scale also between these steps due to Stochasticity in the outcomes. A change from the current to a new correlation was determined probabilistically in every trial with a p = 0.3 transition probability, under the constraint that a change would only occur after the new correlation became theoretically detectable by an ideal observer that was tracking the correlation coefficient in a sliding window over the past five trials. In detail, after the normatively estimated correlation based on the last five trials (similar to the sliding window model below) approached the new generative correlation (with a deviation <0.2), the correlation was allowed to change on all further trials. This prevented overly rapid changes in the generative correlation before subjects could have possibly detected the new correlation coefficient from outcome observations. On average (across subjects and sessions) the correlations changed every ten trials. To discourage subjects from persevering on a more favorable spot of the response scale that would give a reasonable result over a wider range of correlations, and instead be forced to track the correlation explicitly, we further implemented an adaptive rule that if subjects' response was both suboptimal (farther from the optimum than 0.2) and they did not change their response within the past five trials then the correlation would jump to the farthest extreme (either −0.99 or +0.999). This increased the penalty on subjects payout at their current weights and encouraged them to find a better weight allocation. In practice, this constraint came rarely (never for 10 subjects, one or two occurrences in five, and three occurrences in one subject) into use during the fMRI experiment.

### Correlation Learning Model

We modeled trial-by-trial values of the correlation strength by using principles of reinforcement learning (Sutton and Barto, 1998). Reinforcement learning generates in every trial a prediction error as the deviation of the experienced outcome R from the predicted outcome. Those prediction errors, multiplied by the learning rate, are then used to update predictions in future trials:

(1) $$\text{resource}\;\text{value}\text{:}\;{\text{V}}_{\text{i},\text{t}+1}={\text{V}}_{\text{i},\text{t}}+{\text{α}}^{\text{V}}{\text{δ}}_{\text{i},\text{t}},$$

and

(2) $$\text{value}\;\text{prediction}\;\text{error}\text{:}\;{\text{δ}}_{\text{i},\text{t}}\;=\text{R}-{\text{V}}_{\text{i},\text{t}}.$$

The squared prediction error is also a measure of the outcome fluctuation and thereby a quantifier of risk. A sequence of continuously large prediction errors indicates that the outcomes greatly fluctuate, whereby a sequence of small prediction errors indicate that prediction is precise with little deviation. We used this to model the risk h for both resources:

(3) $$\text{resource}\;\text{variance}\text{:}\;{\text{h}}_{\text{i},\text{t}+1}={\text{h}}_{\text{i},\text{t}}+{\text{α}}^{\text{R}}{\text{ε}}_{\text{i},\text{t}},$$

and

(4) $$\text{variance}\;\text{prediction}\;\text{error}\text{:}\;{\text{ε}}_{\text{i},\text{t}}\;={\text{δ}}_{\text{i},\text{t}}^{2}-{\text{h}}_{\text{i},\text{t}}.$$

We then extended this model from independent outcomes to the interaction of outcomes, whereby the product of the individual prediction errors measures the covariation of two outcomes:

(5) $$\text{resource}\;\text{covariance}\text{:}\;{\text{cov}}_{\text{t}+1}={\text{cov}}_{\text{t}}+{\text{α}}^{\text{C}}{\text{ζ}}_{\text{t}},$$

and

(6) $$\text{covariance}\;\text{prediction}\;\text{error}\text{:}\;{\text{ζ}}_{\text{t}}={\text{h}}_{\text{s}1,\text{t}}\;{\text{h}}_{\text{s}2,\text{t}}-{\text{cov}}_{\text{t}}.$$

The correlation coefficient ρ was then defined as the covariance normalized by the individual standard deviations of the two involved outcomes:

(7) $$\text{resource}\;\text{correlation:}\;{\text{ρ}}_{\text{t}}=\frac{{\text{cov}}_{\text{t}}}{\left(\text{sqrt}\left({\text{h}}_{\text{s}1,\text{t}}\right)*\text{sqrt}\left({\text{h}}_{\text{s}2,\text{t}}\right)\right)}.$$

In every trial the correlation coefficient was finally translated into a position on the response slider using the normative function (h_2,t_ – cov_t_)/(h_1,t_ + h_2,t_ – 2 ^∗^ cov_t_), which is derived in the Supplemental Information. This relationship (Figure S1) did not change over the entire course of the experiment (because we always used the same ratio of 1:2 between outcome σ).

We kept the mean of the resource outcomes constant during each session and therefore the optimal strategy was indeed to not update those variables once a reliable estimate had been formed during the observation phase of each block. In fact, the best-fitting learning rate for resource values was consistently very small across subjects (average 0.08), confirming that, as intended by the design, subjects indeed treated the mean as a stable value after the initial observation period and adjusted their learning rate downward to reflect this steady nature (Behrens et al., 2007).

We investigated whether subjects used different learning rates for variance and covariance learning or whether these processes could be described by a single parameter. We did this by comparing a model with separate parameters for variance and covariance learning with a model that used a common parameter for both learning processes. We found that the reduced model could describe learning as well as the full model if model complexity is considered (Table 1). Note that both overall mean value and variance were constant during the experiment but the best-fitting learning rate for variance was higher than for value. This suggests that, in contrast to mean outcome value, subjects continuously updated their estimate of individual risk in response to local temporal fluctuations in the individual variances.

We therefore used the reduced model with a common risk/covariance learning parameter to generate fMRI regressors. Parameter estimates were fit for every individual subject using least-squares minimization between model predicted weights and actual weights set by the subject (see below).

### Alternative Models

We created several alternative models that do not require learning of covariance information. Those models are described in the Supplemental Experimental Procedures.

### Model Comparison

We compared how well each model predicted subjects' behavior by fitting the free parameters of each model such that the mean squared sum of the deviation between model predicted (w^m^) and subjects' weights (w^s^) was minimized. As measure of model fit we then calculated the Bayesian information criterion (BIC) (Schwarz, 1978) as

(8) BIC=2L+kln(n),

and

(9) $$L=\frac{1}{2}n\left(\log\left(2\pi \right)+\log\left(\frac{\sum_{i}^{n}{\left(w_{i}^{s}-w_{i}^{m}\right)}^{2}}{n}\right)+1\right),$$

where *L* is the negative log likelihood function, n = 252 trials and *k* the number of free model parameters (Table 1). We also calculated a generalized r^2^-statistics for each model, which is a standardized measure of model fit analogous to accounted variance (Nagelkerke, 1991). It is computed as $r^{2}=1-L/L_{\mathrm{random}}$.

### Stimuli

Stimuli were presented on a gray background using Cogent 2000 (http://www.vislab.ucl.ac.uk/cogent.php) running in MATLAB. Stimuli were presented using an LCD projector running at a refresh rate of 60 Hz, viewed by subjects via an adjustable mirror.

### FMRI Data Acquisition

Data were acquired with a 3T scanner (Trio, Siemens, Erlangen, Germany) using a 12-channel phased array head coil. Functional images were taken with a gradient echo T2^∗^-weighted echo-planar sequence (TR = 3.128 s, flip angle = 90°, TE = 30 ms, 64 × 64 matrix). Whole brain coverage was achieved by taking 46 slices in ascending order (2 mm thickness, 1 mm gap, in-plane resolution 3 × 3 mm), tilted in an oblique orientation at −30° to minimize signal dropout in ventrolateral and medial frontal cortex (Weiskopf et al., 2006). Subjects' head was restrained with foam pads to limit head movement during acquisition. Functional imaging data were acquired in three separate 415-volume runs, each lasting about 21 min. The first five volumes of each run were discarded to allow for T1 equilibration. A B0-fieldmap (double-echo FLASH, TE1 = 10 ms, TE2 = 12.46 ms, 3 × 3 × 2 mm resolution) and a high-resolution T1-weighted anatomical scan of the whole brain (MDEFT sequence, 1 × 1 × 1 mm resolution) were also acquired for each subject.

### FMRI Data Analysis

Image analysis was performed using SPM8 (rev. 3911; http://www.fil.ion.ucl.ac.uk/spm). EPI images were realigned and unwarped using field maps (Andersson et al., 2001). Each subject's T1 image was segmented into gray matter, white matter, and cerebrospinal fluid, and the segmentation parameters were used to warp the T1 image to the SPM Montreal Neurological Institute (MNI) template. These normalization parameters were then applied to the functional data. Finally, the normalized images were spatially smoothed using an isotropic 8-mm full-width half-maximum Gaussian kernel.

FMRI time series were regressed onto a composite general linear model (GLM) containing regressors representing the time of the choice, the time of the outcome screen, and any button presses during the choice period. The outcome regressor was modulated by a number of model derived decision variables. Modulators for outcome were: prediction errors for the individual resource outcomes and the portfolio outcome (δ_1_, δ_2_, δ_p_), the absolute deviation of the portfolio outcome from the target (|δ_p_|), resource risk (h_1_, h_2_), risk prediction errors (ε_1_, ε_2_), the correlation strength of the resources (ρ), and the correlation prediction error (ζ). A further modulator captured the anticipated magnitude of actual weight updating in the next trial (|w_t_ − w_t+1_|). In contrast to the default procedure in SPM, we entered all regressors and modulators independently (without serial orthogonalization) into the design matrix. Thereby only the additional variance that cannot be explained by any other regressor is assigned to the effect, preventing spurious confounds between regressors (Andrade et al., 1999; Draper and Smith, 1998). Specifically, this ensured that the observed effects of correlation strength and correlation prediction error are solely accountable by effects not explained by signals relating to the variance of individual outcomes.

The regressors were convolved with the canonical HRF, and low frequency drifts were excluded with a high-pass filter (128 s cutoff). Short-term temporal autocorrelations were modeled using an AR(1) process. Motion correction regressors estimated from the realignment procedure were entered as covariates of no interest. Statistical significance was assessed using linear compounds of the regressors in the GLM, generating statistical parametric maps (SPM) of t values across the brain for each subject and contrast of interest. These contrast images were then entered into a second-level random-effects analysis using a one-sample t test against zero.

Anatomical localization was carried out by overlaying the t-maps on a normalized structural image averaged across subjects, and with reference to an anatomical atlas (Duvernoy, 1999). All coordinates are reported in MNI space (Mazziotta et al., 2001). Unless otherwise noted, all statistics are FWE corrected at the cluster level for multiple comparisons at p < 0.05 with a height threshold of p < 0.001 (using the cluster level statistics implementation within SPM). Small volume correction in the outcome variance contrast for striatum was performed within a 12 mm sphere around the seed voxel coordinates (xyz = −10, 3, 3), which were taken from Preuschoff et al. (2006).

### Region of Interest Analysis

We extracted data for all region of interest analyses using a cross-validation leave-one-out procedure: we re-estimated our main second-level analysis 16 times, always leaving out one subject. Starting at the peak voxel for the correlation signal in right insula and for the correlation prediction error in rACC we selected the nearest maximum in these cross-validation second-level analyses. Using that new peak voxel, we then extracted the data from the left-out subject and averaged across voxels within an 8 mm sphere around that peak.

#### Binned Effect Size Plots

To create the effect size plots of the parametric decision variables we first divided the values in our parametric modulator into quartiles and estimating the average BOLD response in relation to each bin. We did this by sorting all trials into four bins according to the magnitude of the model predicted signal and defined the 25th, 50th, 75th, and 100th percentile of the range. Then we created and estimated for each subject a new GLM with four new onset regressors containing the trials of each bin. The parameter estimates of these onset regressors represent the average height of the BOLD response for all trials in that bin. The data plots in Figures 2B and 3B are the average parameter estimates (across all subjects in the cross-validation analyses) converted to percent signal change. This analysis was performed using algorithms in the rfxplot toolbox for SPM (Gläscher, 2009).

#### Covariance/Correlation Comparison

For the test whether bold activity in right insula is better explained by a linear relationship with covariance or correlation we estimated two additional GLMs on BOLD data, each with only one regressor (either model predicted covariance or the correlation coefficient) using Bayesian estimation (Friston et al., 2002). This produced a log-evidence map for each model and we calculated average log evidences across all voxels within our region of interest for every subject and performed a random effects model comparison (Stephan et al., 2009). This analysis suggests that the correlation coefficient can explain BOLD activity in midinsula better than covariance (Dirichlet α = 16.9 for correlation versus 1.1 for covariance; posterior probability [correlation] p = 0.94, exceedance probability ]probability that the correlation model is more likely] ≈1.0).

#### Effect Size Time Course Plots

To visualize the nature of the BOLD response to the correlation coefficient as time course plot over the entire trial we upsampled the entire extracted bold signal to 100 ms (the effective temporal resolution of the averaged time course is higher than the TR because our stimulus presentation was jittered relative to slice acquisition), split the signal into trials and resampled such that the onset of the choice screen is at time 0 and the onset of the outcome screen at 8.5 s in every trial. We then estimated a GLM across trials for every time point in each subject independently. Lastly, we calculated group average effect sizes at each time point, and their standard errors. The graph in Figure 2C shows the time series of effect sizes throughout the trial for the regressor of interest. This method for plotting the effect size time course of a parametrically modulated regressor is also described in detail elsewhere (Behrens et al., 2008).

#### Timing of Correlation Representations

To investigate whether subjects carried out task related computations at the time of the outcome or at the time of choice, we estimated a separate GLM that was similar to the main GLM described above except for an additional parametric modulator at the time of choice for the correlation coefficient, i.e., the correlation coefficient modulated both the regressor at the time of the choice screen and the outcome screen.

#### Representation of Portfolio Weights

We investigated the questions if subjects might learn task-specific portfolio weights instead of the more universal correlation between outcomes by estimating a separate GLM. This was similar to the main GLM except that the parametric modulator ρ was replaced by the portfolio weight w and the correlation prediction error ζ was replaced by the signed weight prediction error (w_t+1_ – w_t_). The nonlinear relationship between ρ and w allows us to differentiate between representations of correlation and weights on the neural level.

#### Representation of Outcome Coincidences

To test for a neural representation of more qualitative coincidences instead of the correlation coefficient with estimated another GLM, similar to the main GLM except that the parametric modulators ρ and ζ were replaced by a binary parametric modulator with a coincidence value of sign(td_1_)^∗^sign(td_2_).

#### Relationship between Explained Variance in Behavioral Model and BOLD Data

To test for a relationship between behavior and neural model fit we compared R^2^ (explained variance) in the behavioral model with the R^2^ in the fMRI GLM. An R^2^ value for the behavioral model was calculated for every subject by regressing trial-by-trial model predicted choice on subject's actual choices. We calculated the R^2^ value for the fMRI regression as the proportion of variance in BOLD that was explained by our interest regressors in relation to the total variance (R^2^ = RSS_reg_/RSS_tot_), where RSS_reg_ equals the explained variance (variance of the predicted time course y^pred^ = Xb, X = design matrix and b the regression coefficient) and RSS_tot_ is the variance of the bold signal after adjusting for block and nuisance effects.

We also tested the influence of potential confounding variables on this relationship, namely the fitted learning rate and the average absolute amount of weight updating per trial, by calculating partial correlations. This analysis confirmed a significant correlation between behavioral and neural fit (r_xy_ = 0.54, p = 0.04) after accounting for potential confounds. Furthermore, there was no relationship between these potential confounds and neural fit (r_ay_ = 0.12, p = 0.66; r_|w|y_ = −0.14, p = 0.63).

#### Psychophysiological Interaction (PPI) Analysis

We performed posthoc an exploratory PPI analysis (Friston et al., 1997) to investigate changes in functional connectivity with right midinsula at the time of outcome (when almost all task related activity was observed). The PPI term was Y × P, with Y being the BOLD time courses in the insula region of interest analysis and P indicating the time during the outcome screen. We then entered the seed region BOLD Y, the psychological variable P, and the PPI interaction term into a new GLM. Findings from this analysis are reported in Figure S4.

## Figures and Tables

**Table 1:** Model Comparison and Model Fit

Model/Parameters	Correlation (var/cov)	Correlation (val/var/cov)	Q-Learning	Coincidence	Sliding Window	1/N	Random Choice
α-Val	0.08	0.08					
α-Risk	0.25	0.25		0.16			
α-Cov	0.25	0.26		0.34			
Learning rate			0.23				
W step width			0.10				
Window length					9.93		
N parameters	2	3	2	2	1	0	0
NLL	87.92	86.20	197.20	142.93	110.96	283.85	384.67
r^2^	0.77	0.78	0.49	0.61	0.71	0.26	0
BIC	186.90	188.99	405.45	296.92	227.44	567.69	769.35
r^2^ forecast	0.77	0.77	0.40	0.56	0.70	0.26	0

Medians of best-fitting parameters for the compared learning algorithms. Parameters were fit to individual subjects across the three scanning blocks. The (pseudo) r^2^ value measures how well the model can capture subjects' behavior (see Experimental Procedures). The r^2^-forecast measure uses a similar normalization to quantify how well the model could estimate the ground truth correlation. To estimate this value, we refit the parameters of each model to estimate ground truth correlations, pooled over all sessions and subjects. BIC, model evidence corrected for complexity (Bayesian information criterion); Cov, covariance; NLL, model evidence (negative log likelihood, smaller is better); Val, value; Var, variance.

**Table 2:** Significant Activations in Statistical Parametric Analysis

	x	y	z	Z	Voxels	p (FWE)	Region	Hemi
Correlation coefficient (ρ)	48	5	−5	4.12	59	0.001^a^	Midinsula	R
	60	−1	−5	3.87	“	“	Midinsula (extending into superior temporal sulcus)	“
	48	−7	−2	3.77	“	“	“	“
	−60	−1	−17	3.85	18	0.61	Superior temporal sulcus	L
	−51	−10	−17	3.20	“	“	“	“
	−18	−16	1	3.56	19	0.44	Thalamus	L
	9	−55	37	4.50	8	0.96	Precuneus	R
	12	−61	−5	3.33	5	0.90	Occipital cortex	L
	−54	−40	4	3.31	4	0.96	Superior temporal sulcus	L
Correlation prediction error (ζ)	−15	44	7	4.87	36	0.003^a^	Rostral ACC	L
	−54	−25	−5	4.01	43	0.14	Superior temporal sulcus	L
	−57	8	−23	3.95	4	0.99	Anterior superior temporal sulcus	L
	−42	−61	37	3.63	17	0.80	Inferior parietal lobe	L
	−60	−1	−14	3.61	10	0.93	Superior temporal sulcus	L
	−63	−7	−8	3.48	“	“	“	“
	12	−13	52	3.57	3	0.91	Medial cingulate gyrus	R
	36	−10	7	3.23	3	0.99	Posterior insula	R
Absolute weight update	6	26	34	4.22	135	0.001^a^	ACC/DMPFC	R
	−9	29	25	3.50	“	“	“	L
	42	23	−5	4.04	55	0.04^a^	Anterior insula	R
	15	−64	34	3.95	40	0.04^a^	Precuneus	R
	51	26	22	3.81	7	0.73	DLPFC	R
	15	−31	26	3.73	15	0.38	Cerebellum	R
	0	−19	−2	3.71	29	0.20	VTA vicinity	
	−33	17	−5	3.57	21	0.69	Anterior insula	L
	−12	2	58	3.37	7	0.88	SMA	L
	0	−52	−35	3.18	6	0.97	Cerebellum	
Risk (average contrast over individual risk from both outcomes, h1/h2)	45	−4	−14	3.69	7	0.76	Posterior insula	R
	45	−76	34	3.67	3	0.98	Posterior parietal cortex	R
	18	−28	4	3.60	3	0.86	Thalamus	R
	−21	2	7	3.55	7	0.85	Striatum	L
	−42	−55	−35	3.38	3	0.99	Cerebellum	L
Risk prediction errors (average contrast over individual risk PE from both outcomes, ε_1_/ε_2_)	−24	23	−8	3.13	3	0.98	Anterior insula	L

ACC, anterior cingulate; DMPFC, dorsomedial prefrontal cortex; FWE, familywise error; Hemi, hemisphere; L, left; MNI, Montreal Neurological Institute; R, right. All peaks are thresholded p < 0.001 uncorrected; listed are all clusters with an extent ≥3 voxels.

a Significant at p < 0.05 FWE cluster level correction in entire brain. Coordinates in MNI space.

## Supplemental Information

Document S1. Four Figures, One Table, Supplemental Experimental Procedures, and Supplemental Text
