# Supplementary material for: Hedging Your Bets by Learning Reward Correlations in the Human Brain
Source: Neuron. 2011 Sep 22;71(6-2):1141–52. doi: 10.1016/j.neuron.2011.07.025 (PMC3183226; doi:10.1016/j.neuron.2011.07.025)
Supplement: Document S1. Four Figures, One Table, Supplemental Experimental Procedures, and Supplemental Text [file mmc1.pdf]

Neuron, Volume *71*

Supplemental Information

Hedging Your Bets by Learning Reward Correlations in the Human Brain

Klaus Wunderlich, Mkael Symmonds, Peter Bossaerts, and Raymond J. Dolan

Inventory of Supplemental Material

Figures S1–S4

Tables S1 and S2

Supplemental Experimental Procedures

Supplemental Text

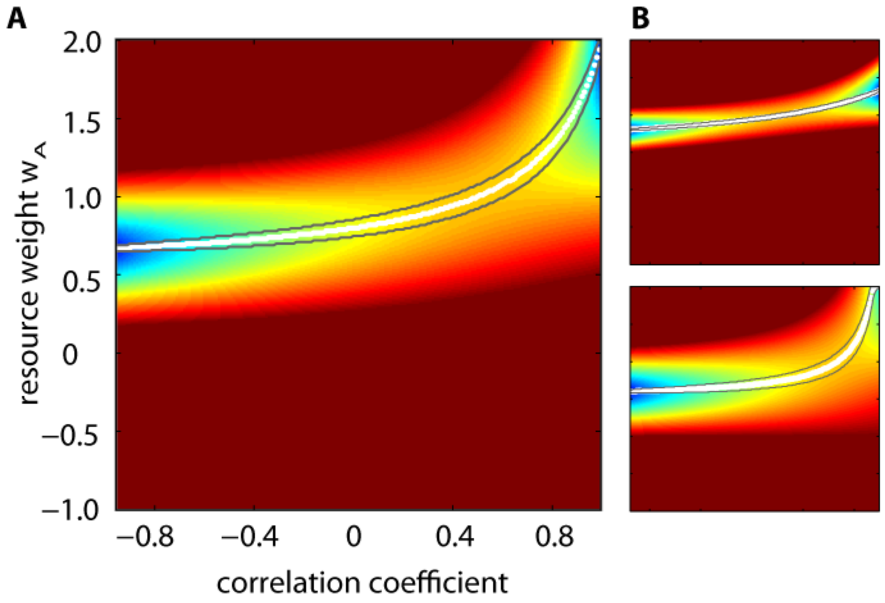

Figure S1. Relationship between Correlation, Optimal Portfolio Weights, and Portfolio Variance

Optimal portfolio weight w_{sun} ($w_{wind} = 1 – w_{sun}$) increases as a function of the correlation coefficient between sun and wind outcomes. The background color indicates portfolio standard deviation (blue = small sd, red = large sd). Optimal portfolio weights (for variance minimization) are displayed as white line, the gray lines indicate the 10% interval around the optimal choice (a deviation of that amount from the optimal weights would result in a 10% higher sd). In the example displayed in (**A**), the sd of resource B is twice the sd of resource A ($\sigma_B = 2*\sigma_A$). In half of the blocks (randomly determined) subjects experienced the opposite relation ($\sigma_B = 1/2*\sigma_A$) and in that case the depicted optimal weight function is mirrored around a horizontal axis at w_A=0.5. This transformation from correlation coefficient to portfolio weights remained constant during the entire experiment. (**B**) Same plot as in A) but with a sd ratio $\sigma_A/\sigma_B = 4$ (top) and $\sigma_A/\sigma_B = 1.5$ (bottom). This plot illustrates that the dynamic range of optimal weights decreases as the ratio increases. In contrast, the steepness at which deviations from the optimum weight influence performance decreases on smaller ratios. The sd ratio of $\sigma_A/\sigma_B = 2:1$ is optimally suited for our experiment as it provides the best tradeoff between dynamic range and specificity.

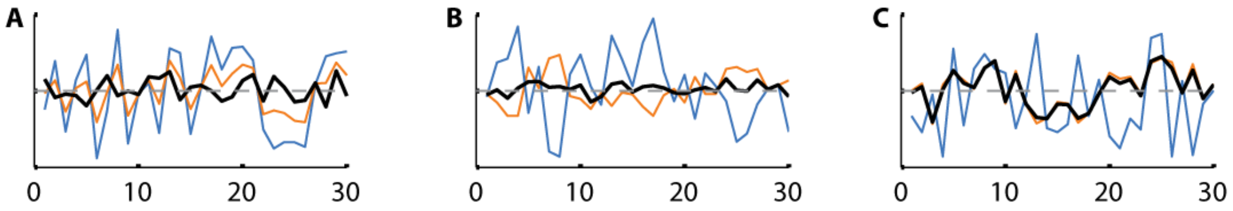

Figure S2. Variance Minimization Strategies

Plotted are example time series for outcome values of resource A (blue) and B (orange) and the combined portfolio outcome value V_p (black). The portfolio is based on MPT calculated weights to minimize variance. Shown are three cases with A and B having (**A**) positive correlation (**B**) negative correlation (**C**) no correlation.

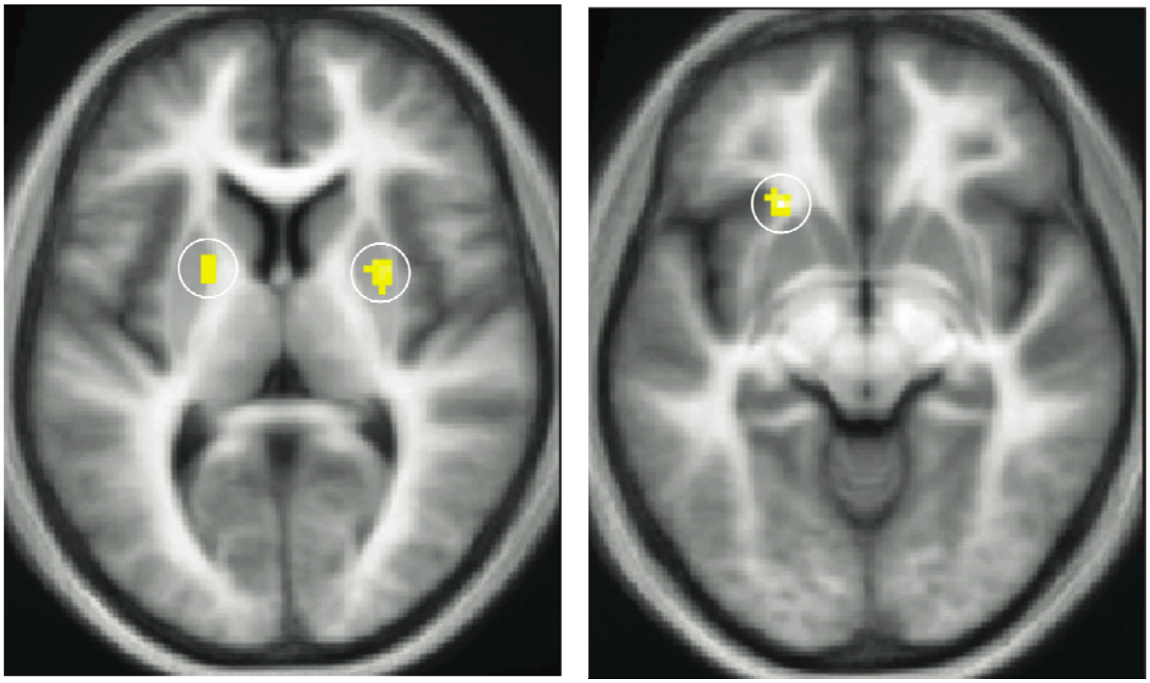

Figure S3. Neural Correlates of Risk

FMRI signals in striatum relate to individual outcome risk (left) and signals in left anterior insula relate to risk prediction error (right). While these results are not a central aspect of our study, they replicate previous findings from (Preuschoff et al., 2006) and confirm that our subjects did indeed form a representation of outcome risk while working on our task. Results in this figure are displayed at a threshold of $p<0.005$ uncorrected.

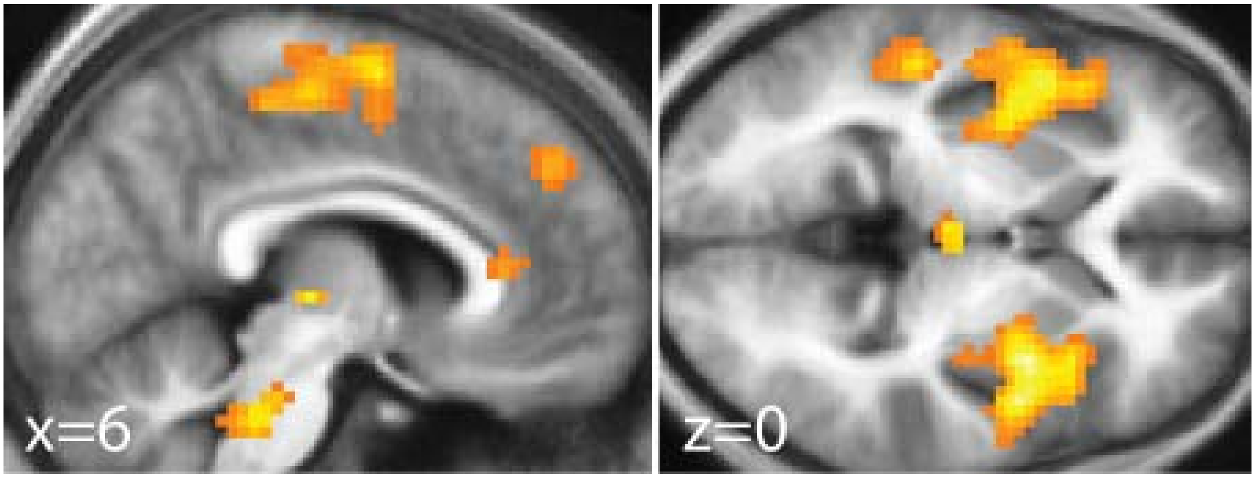

Figure S4. Functional Coupling of Right Medial Insula during Updating of Values

Areas showing increased functional coupling with right medial insula (xyz = 48, 5, -5) at the time of outcome in a physiological/psychological interaction (PPI) analysis. At FWE corrected significance ($p<0.05$ at the cluster level) bilateral insula (xyz = 36, 2, -5; Z=5.13 and -45, 9, -2; X=4.48), bilateral middle frontal gyrus (xyz = -33, -22, 43; Z=4.55 and 36, -19, 46; Z=4.26), bilateral central sulcus (xyz = -21, 31, 64; Z=4.10 and 6, -1, 61; Z=4.02), right enthorinal area (27, -28, -23; Z=4.19), brainstem (xyz = 9, -34, -32; Z=4.03), right angular gyrus (xyz = 60, -58, 31; Z=3.83), and DMPFC (implicated in translating new information into weight updates; xyz = 6, 44, 37; Z=3.52) extending into right superior frontal gyrus (xyz = 15, 47, 40; Z=3.74) showed increased functional coupling. At a lower uncorrected threshold we also observed increased coupling with rostral ACC (implicated in encoding correlation prediction errors; xyz = 6, 32, 7; Z=3.42). Results are displayed at $p<0.001$ uncorrected.

Table S1. Individual Subjects' Performance

The payout bonus benchmarks the portfolio fluctuation realized by the subject against the fluctuation that would result from an optimal strategy (bonus = $sd^{optimal}$ / $sd^{subject}$). The performance index benchmarks subjects' actual responses to optimal responses of an omniscient agent (normalized between 0=random choice and 1=perfect choice).

Subject	*R^2 in behavior on model regression*	*BIC Correlation model*	*BIC Coincidence model*	*BIC Q-Learning model*	*Payout bonus % of maximum*	*Subject performance index*
1	0.88	-11.37	161.25	384.72	0.81	0.67
2	0.84	78.19	149.94	282.01	0.77	0.67
3	0.76	257.58	382.73	488.64	0.82	0.66
4	0.70	289.51	385.76	449.02	0.73	0.5
5	0.64	318.27	362.56	441.13	0.59	0.44
6	0.82	197.48	363.15	489.30	0.76	0.61
7	0.57	263.47	265.03	365.85	0.73	0.46
8	0.77	164.91	258.83	375.37	0.62	0.6
9	0.86	16.18	77.67	353.14	0.81	0.76
10	0.83	207.88	367.55	445.79	0.82	0.66
11	0.79	184.50	327.32	441.23	0.82	0.66
12	0.68	371.54	455.56	529.41	0.64	0.48
13	0.84	105.88	258.71	333.91	0.86	0.75
14	0.89	19.38	263.31	226.11	0.84	0.73
15	0.42	423.82	402.21	490.17	0.74	0.41
16	0.84	103.03	269.14	391.43	0.80	0.64

To make population inferences on model fits, we used Bayesian Model comparison (Stephan et al., 2009) on the behavioral fit data. The exceedance probability in this test provides a likelihood that one model is more likely than the other in the population. This test provides significant evidence for the correlation learning model in all pair wise tests ($p>0.999$), i.e. all other models are less likely than the correlation model.

Supplemental Experimental Procedures

In addition to the correlation learning model described in the main text we created the following alternative models that do not require learning of covariance information.

Model free RL learning

The most basic one in terms of reinforcement learning is model-free RL that learns Q-values for actions (moving the slider right or leftwards, corresponding to increasing or decreasing weights), based on the portfolio outcome. A model free learner would in the beginning start somewhere on the slider (in our modelling we used the center) and then makes an action a_t (a move either left or right). After observing the portfolio outcome he calculates a prediction error as the absolute deviation of this outcome $V_{p,t}$ from the target outcome M (the grand mean of the portfolio outcomes). The Q-value for that action is then updated by the RL-prediction error in the current trial.

$$Q_{a,t} = Q_{a,t} + \alpha\delta^{RL}{}_t \quad (1)$$

$$\text{with } \delta^{RL}{}_t = |(V_p - M)|/100 - Q_{a,t} \quad (2)$$

If the subject experiences a large deviation from the target, then the current move is penalized more than if the subject experiences a small deviation. The Q value for moving in the opposite direction was $1\text{-}Q_a$.

Because the values of the two available actions were directly linked, each outcome provided equal information for both actions. Consistent with the other models we used greedy action selection to determine how weights are updated in every trial (Sutton and Barto, 1998). An additional parameter allowed fluctuations in the step size of resulting weight changes across subjects.

Heuristic based on coincidence detection (outcome – outcome associations)

Instead of learning the relation between two outcomes via their co-variation (a statistical measure related to risk and variance) individuals could form simple associations between one and the other outcome. A subject performing such associative learning would learn an outcome-outcome

association and update the strength of this relation by a trial-by-trial prediction error. This concept is easy to describe for the case of probabilistic outcomes of constant magnitude. If outcome O_1 is present its predictive strength is updated depending on the presence of O_2 as

$$V_{12} = V_{12} + \alpha\,(O_2 - V_{12}) \tag{3}$$

If O_1 is absent, then the strength is inversely updated as

$$V_{12} = V_{12} - \alpha\,(O_2 - V_{12}) \tag{4}$$

This updating strategy increases the associative relationship between O_1 and O_2 whenever the outcomes are both either present or absent and decreases the relationship if only one outcome is present. In our experiment the resource outcomes were always present but fluctuated in their magnitude. To test if subjects nevertheless reduced the problem to mere coincidence detection, we simulated such a strategy by treating resource values greater than the mean as 'present' and below the mean as 'absent'.

We used this model to test whether subjects might take out the quantification of risk from the problem and instead solve the experiment qualitatively by detecting trials in which the two outcomes coincide (a positive relation would result if both are above average or both below average, and a negative if one is above and the other below average). The transformation of outcome association strength (replacing the correlation coefficient) to weight was done similarly as in the full correlation model to allow a direct comparison of the two methods at the learning level.

Sliding window model

A common approach to estimate the local temporal correlation of a time series is to calculate the correlation coefficient over the past n trials. We did this by using a sliding window of size n and calculated the correlation coefficient over these trials:

$$\rho = \frac{\sum (x_i - \bar{x})(y_i - \bar{y})}{\sqrt{\sum (x_i - \bar{x})^2}\sqrt{\sum (y_i - \bar{y})^2}} \tag{5}$$

While this model has little biological plausibility, it is the way of how correlations are often computed in applied mathematics or finance. We therefore include data from the model fit for comparison to the reinforcement learning approach.

The best fitting window size parameter n in a fit of model to subjects' behavior allows us to put the learning rate in the RL algorithm (using an exponential kernel) in relation to a span of observed trials if the correlation was calculated normatively.

1/N Heuristic

This simple heuristic from behavioral finance keeps the weights constant at equal weights for all N assets (N=2 in our case)

$$w_{sun} = w_{wind} = 1/N = 0.5$$

Our task was not a fair test for whether subjects would use the 1/N rule in an uncertain environment because our adaptive design punished the use of constant weights. We nevertheless include parameters from this strategy in our summary table for comparison. The gain from using an optimal strategy over this heuristic decreases with larger N or if the decision maker has very imprecise information about correlations.

Random choice

A random weight (within the range [-1, 2] is chosen on every trial).

Supplemental Text

Variance minimizing strategies in a portfolio

Modern portfolio theory (MPT) is a theory of investment which normatively maximizes expected return for a given amount of risk, or equivalently minimizes risk for a given level of expected return, by solving for the optimal proportions of various assets. It is therefore ideally suited to describe the best possible strategy for setting portfolio weights that minimize overall fluctuation.

The optimal mixing of the two assets depends on their individual variance and their correlation but to simplify our task we kept the mean return of both assets and the standard deviations σ_1 and σ_2 constant with the relationships of either $\sigma_1 = 2*\sigma_2$ or $\sigma_1 = 0.5*\sigma_2$. Hence for the purpose of our experiment the optimal portfolio weight w_1 could be described as a fixed (nonlinear) function of the correlation coefficient. We will explain in the following section how the portfolio variance can be minimized for three cases of a (1) positive correlation, (2) negative correlation and (3) zero correlation.

1) Single trial outcomes from two highly correlated assets tend to deviate from the mean in the same direction and the asset with the larger fluctuation will on average be the one that deviates more. The investor therefore buys assets with the lesser risk and short sells a smaller number of assets with the higher risk. This is realized by a large positive portfolio weight for the outcome that has the smaller variance and a negative weight for the other outcome. Large deviations of the lower risk stimulus are thereby offset by a subtraction of the deviation of the higher risk asset (Fig. S2A).

2) If the two assets are negatively correlated, they tend to deviate from their mean in opposite direction. The portfolio variance is minimal with some form of averaging over both assets (Fig. S2B), i.e. in the optimal solution here the weights for both assets are positive.

3) In a scenario where the assets are truly uncorrelated, the outcome of one reward has no predictive power over the other and their mixing has little effect on variance minimization. In this case the best strategy is to mostly bet on the reward with the lower intrinsic variance.

The relationship between correlation and optimal portfolio weights in our task is depicted as a function in Figure S1.

Optimal weights for minimum portfolio variance

In general form, the portfolio variance is:

$$\sigma_p^2 = \sum_i \sum_j w_i w_j \sigma_i \sigma_j \rho_{ij}$$

For a portfolio with only two assets A and B this is:

$$\sigma_p^2 = w_A^2 \sigma_A^2 + w_B^2 \sigma_B^2 + 2 w_A w_B \sigma_A \sigma_B \rho_{AB} = w_A^2 \sigma_A^2 + (1 - w_A)^2 \sigma_B^2 + 2 w_A (1 - w_A) \operatorname{cov}_{AB}$$

To find the minimum variance weighting we differentiate and solve:

$$\frac{\partial \sigma_p^2}{\partial w_A} = w_A \sigma_A^2 - \sigma_B^2 + w_A \sigma_B^2 + \operatorname{cov}_{AB} - 2 w_A \operatorname{cov}_{AB} \overset{!}{=} 0$$

$$w_A \sigma_A^2 + w_A \sigma_B^2 - 2 w_A \operatorname{cov}_{AB} = \sigma_B^2 - \operatorname{cov}_{AB}$$

$$w_A = \frac{\sigma_B^2 - \operatorname{cov}_{AB}}{\sigma_A^2 + \sigma_B^2 - 2 \operatorname{cov}_{AB}}$$
